# An alarmingly high and increasing prevalence of obesity in Jordan

**DOI:** 10.4178/epih.e2020040

**Published:** 2020-06-06

**Authors:** Kamel Ajlouni, Yousef Khader, Anwar Batieha, Hashem Jaddou, Mohammed El-Khateeb

**Affiliations:** 1 National Center for Diabetes, Endocrinology and Genetics, Jordan University, Amman, Jordan; 2 Department of Public Health, Jordan University of Science and Technology, Irbid, Jordan

**Keywords:** Obesity, Prevalence, Epidemiology, Trend, Jordan

## Abstract

**OBJECTIVES:**

The aim of this study was to determine the prevalence of obesity in Jordan, to assess related trends, and to determine associated factors and comorbidities.

**METHODS:**

A multipurpose national household survey of Jordanian adults was conducted over a 4-month period in 2017. Data were collected using a structured validated questionnaire. Anthropometric measurements including waist circumference (WC; measured midway between the iliac crest and the lower rib margin), body mass index (BMI), hip circumference, waist-to-hip ratio, and waist-to-height ratio were obtained to categorize participants with regard to overweight and obesity.

**RESULTS:**

This study included 4,056 persons (1,193 men and 2,863 women) aged 18 years to 90 years (mean±standard deviation, 43.8±14.2 years). According to the International Diabetes Federation WC criteria, the age-standardized prevalence of obesity was 60.4% among men and 75.6% among women, while approximately three-quarters of men and women were overweight or obese as defined by BMI. The age-adjusted odds of obesity in 2017 were approximately twice those in 2009 in men (odds ratio [OR], 1.98) and women (OR, 1.96). In the multivariate analysis, age, region of residence, and marital status were significantly associated with obesity in both genders. Obesity was significantly associated with increased odds of diabetes mellitus, hypertension, elevated triglycerides, and low high-density lipoprotein cholesterol after adjusting for age.

**CONCLUSIONS:**

The rate of obesity in Jordan is high and increasing, and obesity is associated with other metabolic abnormalities. Well-defined programs to control and prevent obesity, as well as intersectoral action, are urgently required to reverse current trends.

## INTRODUCTION

Obesity is a global public health problem in terms of morbidity, mortality, and its associated economic burden. In 2014, 5.0% of deaths worldwide were attributable to obesity, with an estimated economic impact of approximately 2.8% of the global gross domestic product [1]. Obesity is associated with an increased risk of many chronic conditions, including diabetes, dyslipidemia, stroke, cardiovascular disease (CVD), and certain cancers, and it is also associated with an elevated risk of total mortality and death from CVD [2]. Many anthropometric measures, including waist circumference (WC), body mass index (BMI), hip circumference (HC), waist-to-hip ratio (WHR), and waist-to-height ratio (WHtR), have been used as measures of obesity. These measures have been tested, compared, and used to predict the risk of CVD and other metabolic abnormalities. The question of which measure most accurately reflects body fat distribution is still under considerable debate [3].

The prevalence of obesity has doubled since 1980 in more than 70 countries, and obesity affected 107.7 million children and 603.7 million adults worldwide in 2015 [4]. Almost half of the world’s adult population is predicted to be overweight or obese by 2030 [5]. The prevalence rates of obesity are alarmingly high and increasing in many countries in the Eastern Mediterranean Region (EMR) due to changes in food consumption, reduced physical activity, and an increasingly sedentary lifestyle [6,7]. In adults, the prevalence of obesity increased from 15.1% in 1980 to 20.7% in 2015 [8]. The contribution of high BMI to total disability-adjusted life years (DALYs) in the EMR increased from 3.7% of DALYs in 1990 to 7.5% of DALYs in 2013 [9].

A survey in 2008 showed that the age-standardized prevalence rate of obesity among adults in northern Jordan was 28.1% in men and 53.1% in women. That study also documented an increased rate of obesity in the 10 years preceding the survey [10]. This increase in obesity coincided with increased rates of diabetes, hypertension, and dyslipidemia in Jordan [11-13]. Ten years later, a representative survey using a larger sample size was conducted in the same country. This study aimed to determine the prevalence of obesity in Jordan, to assess related trends, and to determine the associated factors and comorbidities.

## MATERIALS AND METHODS

### Study design and sampling

A multipurpose national household survey was conducted among Jordanian adults over a period of 4 months in 2017. A multistage sampling technique was used to select a nationally representative sample from the population of Jordan. A village or city was selected from each of the 12 governorates of Jordan. The sample of households was chosen in 2 stages. In the first stage, well-defined geopolitical areas were selected from each village or city. The second stage of household selection involved choosing a random sample of households using a systematic sampling technique in each selected area. A team of 2 people (1 woman and 1 man) visited the selected households, explained the study, and invited the members of each household to report to the health center in the selected area after fasting all day. Subjects were asked not to take their medications that day and to bring their medications with them to the health center. Subjects aged ≥ 18 years were eligible for inclusion in the study.

### Data collection

Data were collected using a structured, validated, and pilot-tested questionnaire administered by trained interviewers. The questionnaire was identical to one used in a 2009 survey. This questionnaire included items intended to assess the socio-demographic variables and clinical characteristics of participants, including selfreported diagnoses and treatment of diabetes and hypertension. Three blood samples were drawn from a cannula inserted into the antecubital vein and used for the laboratory measurements. Tubes containing sodium fluoride potassium oxalate were used for the blood glucose measurement. Samples were centrifuged within 1 hour at the survey site and transferred in separate labeled tubes in ice boxes to the central laboratory of the National Center of Diabetes, Endocrinology, and Genetics in Amman, Jordan. All biochemical measurements were carried out by the same team of laboratory technicians using the same method. Fasting plasma glucose was measured via the glucose oxidase method using a Roche cobas analyzer (F. Hoffmann-La Roche Ltd., Basel, Switzerland).

### Anthropometric measurements

Several different anthropometric measures—WC, BMI, WHR, and WHtR—were calculated to define overweight and obesity. Weight was measured with subjects minimally clothed without shoes using digital scales (Seca GmbH, Hamburg, Germany). Height was measured using a portable stadiometer (Seca 214, Seca GmbH). BMI was calculated as weight in kilograms divided by height in meters squared. WC was measured midway between the iliac crest and the lower rib margin, over light clothing and using unstretchable tape (Seca 203, Seca GmbH), without exerting any pressure on the body surface. WHR was calculated as WC divided by HC, and WHtR was calculated as WC divided by height in centimeters. All measurements were taken by the same team of well-trained persons using the same tools.

### Variable definitions

Overweight and obesity were defined using various criteria and standards. Based on BMI, overweight was defined as a BMI ≥ 25 kg/m^2^ and < 30 kg/m^2^, while obesity was defined as a BMI ≥ 30 kg/m^2^. Based on WC, obesity was defined using the Adult Treatment Panel III criteria (WC ≥ 88 cm for women and ≥ 102 cm for men) and separately using the International Diabetes Federation (IDF) criteria. As recommended by the IDF criteria, the European cut-off values were used to define obesity among Jordanian adults (WC ≥ 80 cm for women and ≥ 94 cm for men). The World Health Organization criteria (WHR > 0.85 for women and > 0.90 for men) were used to define obesity based on WHR. Participants were considered to have recently diagnosed diabetes if they had fasting blood sugar ≥ 126mg/dL (≥ 7.0mmol/L) at the time of the survey with no prior history of diabetes. Participants were considered to have recently diagnosed hypertension if they had a systolic blood pressure of ≥ 140 mmHg and/or a diastolic blood pressure of 90 mmHg at the time of the survey with no prior history of hypertension. Previous diagnoses of diabetes and hypertension were self-reported by participants. Metabolic risk factors were defined using the 2006 IDF criteria, which defined elevated triglycerides as ≥ 150 mg/dL (≥ 1.7 mmol/L) and reduced high-density lipoprotein (HDL) cholesterol as < 40 mg/dL (< 1.03 mmol/L) for men and as < 50 mg/dL (< 1.29 mmol/L) for women.

### Statistical analysis

Data were entered into and analyzed using SPSS version 20 (IBM Corp., Armonk, NY, USA). Data were described using means and percentages. To permit comparison between the different surveys and with studies in other countries, age-standardized prevalence rates of obesity were derived using the world population as a standard. Additionally, 95% confidence limits were reported for these standardized rates. The chi-square test and crosstabs analysis were used to compare differences between proportions. To assess the changes in the likelihood of obesity between the 2009 and 2017 surveys, the data of both surveys were merged and analyzed after adjusting for age. Binary logistic regression analysis was conducted to determine factors associated with obesity, while other binary logistic regression models were utilized to evaluate the associations between obesity and metabolic abnormalities. A p-value < 0.05 was considered to indicate statistical significance.

### Ethics statement

The study was approved by the Ethical Committee at the National Center for Diabetes, Endocrinology, and Genetics in Amman, Jordan. Informed consent was obtained from each participant, and data were treated with strict confidentiality and used only for scientific purposes.

## RESULTS

### Participants’ characteristics

This study included a total of 4,056 persons (1,193 men and 2,863 women). Their ages ranged from 18 years to 90 years, with a mean± standard deviation of 43.8± 14.2 years. The demographic and clinical characteristics of the participants according to gender are shown in Table 1. Approximately 41.6% of men and 27.8% of women were older than 50 years. Men were significantly more likely than women to have diabetes, hypertension, elevated triglycerides, and low HDL cholesterol. Table 2 shows the means of the anthropometric and clinical parameters for men and women. While men had significantly higher mean WC and WHR, women had significantly higher mean HC, WHtR, and BMI. The mean values of systolic blood pressure, diastolic blood pressure, triglycerides, and fasting blood glucose were significantly higher in men, while the mean values of total cholesterol and HDL cholesterol were significantly higher among women.

### Prevalence of obesity

Table 3 shows the crude and age-standardized prevalence rates of obesity for men and women using various definitions of obesity. Within each gender, the prevalence varied according to the definition used. According to IDF criteria, the age-standardized prevalence of obesity was 60.4% among men and 75.6% among women. As defined by BMI, approximately three-quarters of both men and women were overweight or obese. Finally, using the recommended measure of WHtR for the Jordanian population, 44.2% of men and 47.8% of women were determined to be obese.

Table 4 shows the prevalence of WC-defined obesity in men and women according to relevant demographic characteristics. In both genders, the prevalence of obesity increased significantly with age. The prevalence varied significantly according to the region of residence and was the lowest in the central region for both men and women. The prevalence did not significantly differ according to the type of residential area (urban vs. rural) in men or women. The variation in the prevalence of obesity according to smoking status was significant among men only.

### Changes in the prevalence of waist circumference-defined obesity between 2009 and 2017

**Figure 1.**
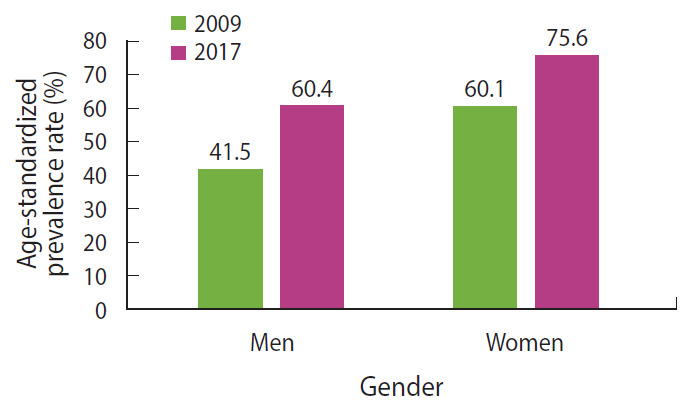
Age-standardized prevalence rates of obesity (defined according to International Diabetes Federation standards) by gender in 2009 and 2017 surveys.

The data from the 2009 and 2017 surveys were merged and analyzed to assess the changes in the likelihood of obesity as defined by WC (per the IDF criteria). Figure 1 shows the age-standardized prevalence rates of obesity among men and women in 2009 and 2017 surveys. After adjusting for age, the odds of obesity in 2017 were twice the odds in 2009 in both men (OR, 1.98; 95% confidence interval [CI], 1.61 to 2.35) and women (OR, 1.96; 95% confidence interval, 1.73 to 2.22). In both survey years, the odds of obesity increased with increasing age, peaked at 60-69 years old in both genders, and declined thereafter.

### Factors associated with waist circumference-defined obesity

In the multivariate analysis, age, place of living, and marital status were found to be significantly associated with obesity as defined by WC (per the IDF criteria) among both men and women (Table 5). Older men and women had higher odds of obesity than younger individuals. Compared to men living in the north, those living in the central region had lower odds of obesity, while those living in the south had higher odds. Compared to women living in the north, those living in the central region had lower odds of obesity. Married people had significantly higher odds of obesity than single individuals for both genders. Current smoking among men was significantly associated with decreased odds of obesity relative to non-smokers. In men and women, the odds of obesity decreased significantly as the number of hours spent per week performing hard physical activity increased. The odds of obesity among men increased by 4% for each additional daily meal.

### Metabolic abnormalities associated with waist circumference-defined obesity

Table 6 shows the metabolic abnormalities associated with obesity as defined by WC (according to IDF criteria) among men and women after adjusting for age. Obesity defined in this manner was found to be significantly associated with increased odds of diabetes mellitus (ORs, 2.1 and 2.9 for men and women, respectively), hypertension (ORs, 2.4 and 2.5 for men and women, respectively), elevated triglycerides (ORs, 2.5 and 4.2 for men and women, respectively), and low HDL (ORs, 2.2 and 2.1 for men and women, respectively) after adjusting for age.

## DISCUSSION

This study demonstrated that the prevalence of obesity in Jordan is alarmingly high in both genders, regardless of the definition of obesity used. Comparing the prevalence of obesity between countries is challenging due to differences in the sampling approaches utilized, age groups included, and anthropometric measures used to define obesity. Since controversy exists regarding which measure most accurately reflects body fat distribution, we used various measures for obesity in the present study. Furthermore, many previous studies in the EMR region have failed to report standardized rates, which makes comparisons more difficult. Using different measures of obesity and reporting age-standardized estimates would facilitate comparisons between the findings of this study and those of other studies.

The rate of obesity in Jordan is among the highest in the region. High rates have also been reported in other EMR countries, including Saudi Arabia [14] and Kuwait [15]. Half of the global population is predicted to be overweight or obese by 2030 [16]. In 2015, the prevalence of obesity in the countries of the Organization for Economic Cooperation and Development ranged from less than 6% in Korea and Japan to greater than 30% in Hungary, New Zealand, Mexico, and the United States [17]. In 2015, Chooi et al. showed that the prevalence of obesitywas >20% in many countries, including Germany (20.9%), Brazil (22.6%), Argentina (23.2%), Russia (24%), the United Kingdom (24.3%), Turkey (28.5%), Mexico (28.6%), South Africa (30.8%), Iraq (31.9%), the United States (33.6%), and Egypt (35.3%) [18]. The prevalence rates of obesity in the United States and the United Kingdom remained at around 30-34% and 23-24%, respectively, between 2005 and 2015 [18]. China witnessed a nearly 9-fold increase in the prevalence of obesity over the past 35 years, from 0.6% in 1980 to 5.3% in 2015 [19].

Per the findings of a previous survey in 2009, the odds of obesity in Jordan in 2017 were approximately twice the odds in 2009 in both men and women after adjusting for age. Lifestyle changes, including the growing consumption of Western-style fast food, reduced physical activity, and an increasingly sedentary lifestyle may explain the increasing rate of overweight and obesity. A similarly increasing trend for obesity has been seen in many countries of the region. The mean BMI and the prevalence of obesity among adults in the EMR increased between 1980 and 2015, possibly due to social and demographic transitions in the area as well as lifestyle changes [6,9].

The prevalence of obesity, even when assessed using different indicators, was found to be greater among women than men in this study. The higher rates among women might be explained by traditional/cultural restrictions in lifestyle choices available to women and limited access to exercise.

The present study showed that married people were more likely to be obese than unmarried individuals. A similar finding was reported in another study in Jordan, which found that the prevalence rates of obesity in married and unmarried adults were 54% and 37%, respectively [10]. A study in Syria yielded similar results, with obesity prevalence rates of 45% and 21% among married and unmarried adults, respectively [20]. Other studies in the region [21-23] have reported similar findings. Marital status has been found to have a mixed relationship with obesity, and the mechanism behind this relationship is poorly understood. The higher odds of obesity among married participants in the present study may be explained by decreases in physical activity, changes in dietary patterns, and a potentially decreased focus on being attractive among married individuals [24-26]. In contrast, unmarried people may pay closer attention to their weight in an effort to appear more attractive to potential marital partners. In married individuals, living in a common household provides social support and can create a responsibility to eat together; this may cause married people to eat more regularly, leading to weight gain [25,27]. This is also supported by our finding that the odds of obesity among men increased by 4% for each additional daily meal.

The rate of obesity was lower in the central region, where people tend to be higher in socioeconomic status, than in the northern and southern regions. The differences in the prevalence of obesity according to the region of residence may reflect the effects of education level and socioeconomic status on obesity. People with a higher educational level and a higher socioeconomic status may be more concerned about their health and have more positive attitudes toward healthy lifestyles than those with less education or a lower socioeconomic status [27].

In this study, smoking was found to be inversely associated with obesity. This finding is consistent with the results of a number of studies [28,29], although the opposite has also been reported [30]. The relationship between smoking and obesity is complex and not fully understood, and published studies have produced conflicting results.

In conclusion, the rate of obesity in Jordan is high and increasing, and obesity in Jordan is associated with other metabolic abnormalities. If this increase in the prevalence of obesity continues, it could lead to serious health-related outcomes and consequences. Well-defined programs to manage, control, and prevent obesity, as well as intersectoral action, are urgently required to reverse current trends in Jordan.

## Figures and Tables

**Table 1.** Demographic and clinical characteristics of participants

Characteristics	Men	Women	Total (n)	p-value
Age (yr)				<0.001
<40	335 (28.1)	1,208 (42.3)	1,543	
40-50	361 (30.3)	855 (29.9)	1,216	
>50	495 (41.6)	793 (27.8)	1,288	
Region				<0.001
Northern	390 (32.7)	922 (32.2)	1,312	
Central	471 (39.5)	1,295 (45.2)	1,766	
Southern	332 (27.8)	646 (22.6)	978	
Type of residential area				0.023
Rural	88 (7.4)	275 (9.6)	363	
Urban	1,105 (92.6)	2,588 (90.4)	3,693	
Marital status				
Single	144 (12.1)	462 (16.1)	606	0.001
Married	1,049 (87.9)	2,401 (83.9)	3,450	
Nationality				
Jordanian	1,117 (94.0)	2,669 (93.9)	3,786	0.893
Syrian	71 (6.0)	173 (6.1)	244	
Smoking				<0.001
None	592 (49.6)	2,628 (91.8)	3,220	
Past	206 (17.3)	46 (1.6)	252	
Current	395 (33.1)	189 (6.6)	584	
Previously diagnosed diabetes	326 (34.6)	433 (18.8)	759	<0.001
Previously diagnosed hypertension	336 (39.2)	614 (26.6)	950	<0.001
Elevated triglycerides	646 (54.6)	1,036 (36.5)	1,682	<0.001
Low HDL	727 (61.4)	1,649 (58.2)	2,376	0.057
High blood pressure or on antihypertensive medication	647 (54.4)	1,079 (38.2)	1,726	<0.001
High fasting blood sugar (>100 mg/dL) or on antidiabetic medication	560 (47.4)	940 (33.3)	1,500	<0.001
Physical activity (hr/wk)				
Light	12.7±14.9	7.5±10.1		<0.001
Moderate	12.2±17.7	13.3±14.2		<0.001
Hard	4.4±10.8	1.8±4.8		<0.001
Consumed vegetables at least 3 times a day (d/wk)	2.4±2.4	3.0±2.7		<0.001

Values are presented as number (%) or mean±standard deviation. HDL, high-density lipoprotein.

**Table 2.** Anthropometric and clinical parameters by gender

Variables	Men	Women	p-value
Waist circumference (cm)	98.9±15.1	92.7±16.6	<0.001
Hip circumference (cm)	104.7±11.4	108.3±13.9	<0.001
Waist-to-hip ratio	0.94±0.09	0.86±0.11	<0.001
Waist-to-height ratio	0.57±0.09	0.59±0.11	<0.001
Body mass index (kg/m^2^)	28.4±4.8	30.0±6.4	<0.001
SBP (mmHg)	127.1±19.6	118.3±19.7	<0.001
DBP (mmHg)	79.5±11.7	75.0±11.6	<0.001
Total cholesterol (mg/dL)	193.3±45.8	197.5±41.7	0.004
HDL cholesterol (mg/dL)	38.2±9.5	48.6±12.3	<0.001
LDL cholesterol (mg/dL)	125.0±37.3	126.9±36.3	0.135
Triglycerides (mg/dL)	203.8±209.3	147.3±110.6	<0.001
Blood sugar (mg/dL)	114.3±48.6	103.1±35.9	<0.001

Values are presented as mean±standard deviation. SBP, systolic blood pressure; DBP, diastolic blood pressure; HDL, high-density lipoprotein; LDL, low-density lipoprotein.

**Table 3.** Age-standardized prevalence rates of obesity by gender using different definitions of obesity

Obesity indicator	Men		Women	
	Crude prevalence, %^1^	Standardized rate, % (95% CI)	Crude prevalence, %^1^	Standardized rate, % (95% CI)
Body mass index ≥25 kg/m^2^	77.3	77.2 (69.4, 74.9)	77.3	74.5 (72.9, 76.0)
Waist circumference (IDF criteria)	67.3	60.4 (57.6, 63.2)	77.8	75.6 (74.2, 77.0)
Waist circumference (ATP III criteria)	41.5	36.5 (33.8, 39.3)	62.8	60.7 (59.1, 62.2)
Waist-to-hip ratio (WHO criteria)	71.8	63.4 (60.6, 66.0)	48.5	47.5 (45.8, 49.1)
Waist-to-height ratio (Jordan-specific cut-off values)	52.1	44.2 (41.5, 46.9)	49.4	47.8 (46.2, 49.3)

CI, confidence interval; IDF, International Diabetes Federation; ATP III, Adult Treatment Panel III; WHO, World Health Organization.

1 Men: 41.2% overweight and 36.1% obese; Women: 29.1% overweight and 48.2% obese.

**Table 4.** Prevalence of waist circumference-defined obesity by gender according to relevant demographic characteristics

Variables	Men	p-value	Women	p-value
Age (yr)		<0.001		<0.001
20-29	41 (26.6)		244 (42.3)	
30-39	114 (64.4)		447 (72.4)	
40-49	230 (73.5)		665 (88.2)	
50-59	222 (76.0)		510 (94.1)	
60-69	119 (77.8)		244 (99.2)	
≥70	71 (74.7)		83 (98.8)	
Region		<0.001		<0.001
Northern	258 (66.5)		753 (82.4)	
Central	280 (59.6)		921 (71.2)	
Southern	259 (79.2)		524 (84.5)	
Type of residential area		0.555		0.287
Rural	61 (70.1)		220 (80.3)	
Urban	736 (67.0)		1978 (77.5)	
Marital status		<0.001		<0.001
Single	40 (28.2)		197 (43.2)	
Married	757 (72.6)		2,001 (84.4)	
Smoking status		<0.001		0.360
None	406 (69.0)		2,010 (77.5)	
Past	168 (82.4)		39 (84.8)	
Current	223 (56.7)		149 (80.1)	

Values are presented as number (%).

**Table 5.** Factors associated with obesity as defined by increased waist circumference (using the IDF criteria) in the multivariate analysis

Variables	Men	p-value	Women	p-value
Age (yr)				
<40	1.0 (reference)		1.0 (reference)	
40-50	2.0 (1.3, 2.9)	0.001	4.2 (3.3, 5.5)	<0.001
>50	2.3 (1.6, 3.4)	<0.001	15.0 (9.8, 22.9)	<0.001
Region				
Northern	1.0 (reference)		1.0 (reference)	
Central	0.7 (0.5, 1.0)	0.029	0.5 (0.4, 0.6)	<0.001
Southern	2.1 (1.4, 3.1)	<0.001	1.0 (0.7, 1.4)	0.939
Marital status (married vs. single)	3.8 (2.4, 6.2)	<0.001	4.3 (3.3, 5.5)	<0.001
Smoking				
None	1.0 (reference)			
Past	1.5 (1.0, 2.3)	0.066	-	
Current	0.6 (0.4, 0.8)	<0.001	-	
Hard physical activity (hr/wk)	0.98 (0.97, 0.99)	0.005	0.97 (0.96, 0.99)	0.003
Average no. of daily meals	1.04 (1.01, 1.08)	0.017	-	

Values are presented as odds ratio (95% confidence interval). IDF, International Diabetes Federation.

**Table 6.** Metabolic abnormalities associated with increased waist circumference by gender after adjusting for age

Variables	Men	p-value	Women	p-value
Diabetes mellitus	2.1 (1.6, 2.8)	<0.001	2.9 (2.1, 3.9)	<0.001
Hypertension	2.4 (1.8, 3.1)	<0.001	2.5 (1.9, 3.3)	<0.001
Elevated triglycerides	2.5 (1.9, 3.2)	<0.001	4.2 (3.1, 5.6)	<0.001
Low HDL	2.2 (1.7, 2.8)	<0.001	2.1 (1.7, 2.5)	<0.001

Values are presented as odds ratio (95% confidence interval). HDL, high-density lipoprotein.
